# 外泌体分离富集技术及其在疾病诊疗中的应用

**DOI:** 10.3724/SP.J.1123.2024.09007

**Published:** 2025-05-08

**Authors:** Guoshan HOU, Huiming YUAN, Zhen LIANG, Lihua ZHANG, Yukui ZHANG

**Affiliations:** 1.中国科学院分离分析化学重点实验室, 中国科学院大连化学物理研究所, 辽宁 大连 116023; 1. CAS Key Laboratory of Separation Sciences for Analytical Chemistry, Dalian Institution of Chemical Physics, Chinese Academy of Sciences, Dalian 116023, China; 2.中国科学院大学, 北京 100049; 2. University of Chinese Academy of Sciences, Beijing 100049, China

**Keywords:** 外泌体, 分离富集, 疾病诊疗, exosome, isolation and enrichment, disease diagnosis and treatment

## Abstract

外泌体是由细胞分泌的脂质双分子层包裹的纳米级囊泡颗粒,携带多种蛋白质、脂质、核酸、代谢物等成分,广泛存在于各种体液中,是细胞间通讯的重要介质。外泌体参与免疫调节、血管生成、肿瘤发生和转移等多种生理病理过程,在临床诊疗中具有巨大潜力。首先,外泌体来源丰富、结构稳定、反映亲本细胞状态,有望成为疾病的新型诊断标志物。其次,干细胞来源的外泌体具有治疗潜力,且具有低免疫原性、高安全性和易于存储等优点,对神经退行性疾病、心血管疾病和癌症等疾病具有治疗潜力。另外,外泌体生物相容性好,具有天然的归巢性能,易穿透生物屏障,是优良的药物递送载体。外泌体的分离富集是其下游分析和应用的前提,高纯度、高收率、高通量的分离方法将有助于发挥外泌体在临床诊疗中的应用潜力。基于外泌体的密度、尺寸、电荷、表面成分等物理化学性质,外泌体分离方法主要分为基于密度的分离方法、基于尺寸的分离方法、聚合物沉淀法以及化学亲和法等。本文对目前外泌体分离富集技术的最新进展进行了归纳总结,并对其在疾病诊断标志物筛选、干细胞来源外泌体用做治疗剂以及外泌体用做药物递送载体等应用进行了介绍,最后对面向疾病诊疗的外泌体分离富集技术的未来发展趋势进行了展望。

## Abstract (en)

Exosomes are nanoscale vesicles wrapped in lipid bilayers that are secreted by cells and carry a variety of proteins, lipids, nucleic acids, and metabolites. Exosomes are widely present in various bodily fluids and mediate intercellular communication. They participate in a variety of physiological and pathological processes, including immune regulation, angiogenesis, tumorigenesis, and metastasis, and have significant clinical diagnosis and treatment potential. Exosomes are source-rich, structurally stable, and reflect the states of their parental cells. Therefore, they are expected to serve as novel diagnostic markers for various diseases. In addition, stem-cell-derived exosomes show therapeutic potential and have the advantages of low immunogenicity, high safety and easy storage, and exhibit therapeutic potential for neurodegenerative disorder, cardiovascular disease, and cancer. Furthermore, exosomes are highly biocompatible, have natural homing properties, and are capable of easily penetrating biological barriers, making them excellent drug-delivery carriers. Isolation and enrichment of exosomes is a prerequisite for downstream analysis and application. High-purity, high-yield, and high-throughput exosome-isolation methods are expected to be used in clinical diagnosis and treatment applications. Based on the physicochemical properties of exosomes, including density, size, charge, and surface composition, exosome-isolation methods are mainly divided into density-based (e.g., differential ultracentrifugation, density-gradient ultracentrifugation), size-based (e.g., ultrafiltration, size-exclusion chromatography, field-flow fractionation), polymer-precipitation (e.g., polyethylene-glycol-based precipitation), and chemical affinity (e.g., antibody-based, aptamer-based, and surface-lipid-based lipid probes) methods. Currently, basic research into exosomes and their clinical applications face a number of challenges. Firstly, the complexity and heterogeneity of exosomes and the lack of standardized isolation methods has led to highly variable research results that hinder comparing and reproducing results between different laboratories and clinical settings. Current isolation methods are generally hindered by insufficient purity, low yield, low throughput, and difficulties separating specific subpopulations, which seriously restrict the development of the exosome field. Secondly, exosome-isolation methods that are easy to use in the clinic, have few technical requirements, and are highly efficient and inexpensive are lacking. Commonly used classical methods, such as ultracentrifugation, are time-consuming, labor-intensive, require large sample volumes, and are inappropriate for clinical settings. Methods such as immunoaffinity can be used to isolate exosomes from precious trace samples in clinical practice; however, high costs, low recoveries, and high operating requirements are shortcomings that restrict sample analysis in the clinic. In addition, robust large-scale methods for preparing exosomes are lacking. There is an urgent need to develop repeatable and scalable methods for preparing batches of high-quality exosomes owing to the rapid development of exosomes for the treatment of clinical diseases. Generally, exosome research progress is expected to greatly improve our understanding of the biological functions and components of exosomes, which will help transform the exosome research into effective diagnostic and therapeutic strategies and lead to new precision-medicine and personalized-treatment applications. This article summarizes the latest progress in exosome-isolation and -enrichment technologies and introduces the application of exosomes as disease diagnostic markers, therapeutic agents, and drug delivery carriers. Finally, the future developmental trends in exosome isolation and enrichment technologies for disease diagnosis and treatment are discussed.

外泌体是由细胞分泌的脂质双分子层包裹的纳米级囊泡结构,携带多种蛋白质、脂质、核酸、代谢物等成分,广泛存在于血液、尿液、唾液、母乳、脑脊液和泪液等体液中^[1]^。外泌体来自母体细胞,可与受体细胞进行物质和信息交流,成为细胞间通讯的重要介质,参与免疫调节、血管生成、肿瘤发生和转移等多种生理病理过程^[2,3]^。由于其来源丰富、结构稳定、反映亲本细胞状态,外泌体作为疾病的诊断标志物在液体活检中具有巨大潜力^[4]^。此外,研究发现干细胞分泌的外泌体具有治疗潜力,但相比于干细胞,外泌体不会出现异常分化和肿瘤生长等问题,且具有高生物活性、低免疫原性、高安全性和易于存储等优点,有望成为神经退行性疾病、心血管疾病和癌症等疾病的新型治疗剂^[5]^。另外,外泌体生物相容性好,具有天然的归巢性能,易穿透生物屏障(例如血脑屏障),可被修饰靶向特定细胞,且能装载多种药物,是优良的药物递送载体^[6]^。因此,外泌体在疾病的诊断、治疗和药物递送等方面展现出良好的应用前景。

外泌体的分离富集是其下游分析和应用的前提。但外泌体本身粒径小(30~200 nm),含量低,在粒径、内容物、功能和来源方面具有高度异质性,且存在于含有各种干扰物的复杂体液微环境中,这使得外泌体的高效分离成为挑战^[1]^。外泌体分离技术主要基于外泌体自身的密度、大小、电荷等物理特性以及表面蛋白质、脂质等化学成分的特异性亲和,实现外泌体与其他成分的区分^[7]^。纯度、回收率、通量等是衡量一项外泌体分离技术是否先进的基本指标。随着近十几年外泌体领域的高速发展,新的外泌体分离方法层出不穷,但目前依然没有单一方法能够兼顾高纯度、高回收率、高通量,且能够保持外泌体高生物活性。因此,仍需要发展新型的外泌体分离制备方法,既可实现高质量外泌体的规模化制备以满足临床治疗应用,又可实现微量样品中外泌体的高效分离以满足临床微量珍贵样品的分析检测。

本文对目前外泌体分离技术的最新进展进行了归纳总结,并对外泌体作为疾病诊断治疗标志物、疾病治疗剂、药物递送载体等在临床诊疗研究中的应用进行了介绍,最后进行了总结和展望,以期为外泌体分离新方法和新技术的开发提供参考。

## 1 外泌体分离富集

外泌体的分离和富集是其生物医学研究和临床转化的必要前提。目前在外泌体研究领域没有标准的分离方法,许多分离方法存在耗时耗力、分离效率低、影响生物活性等不足,限制了它们对临床环境的适用性。制约外泌体分离富集的主要技术障碍在于:(1)如何区分外泌体与其他干扰成分,例如尺寸为200~1000 nm的微泡以及在尺寸、密度上和外泌体有重叠的脂蛋白等;(2)如何简化外泌体的提取,提高外泌体的纯度、产率、回收率和通量,并且能够保持外泌体的高生物活性。基于外泌体的密度、尺寸、电荷、表面成分等物理化学性质,外泌体分离富集方法可以分成以下几类:基于密度的分离方法、基于尺寸的分离方法、聚合物沉淀法以及化学亲和法等。本节将对传统及新兴的外泌体分离富集方法的最新进展进行综述。

### 1.1 基于密度的分离方法

基于密度的分离方法主要根据外泌体与杂质的沉降系数的差别,利用高速离心力对外泌体进行分离富集,可分为差速超速离心法和密度梯度离心法两类。

#### 1.1.1 差速超速离心法

1987年,Johnstone等^[8]^首次采用差速超速离心技术从绵羊网织红细胞培养基中分离出外泌体,目前该法已成为外泌体分离最常使用的“金标准”。该技术基于外泌体的沉降系数与其他共存污染物的差异,在300 g离心力去除细胞、2000 g离心力去除死细胞、10000 g离心力去除细胞碎片后,以100000 g离心力对外泌体颗粒进行沉降,实现外泌体的获取^[9]^。差速超速离心法分离外泌体的效率受到离心参数(如转子类型、转速和持续时间)和样品性质(如黏度和体积)等因素的影响,实际操作过程中需考虑这些因素以获得最佳的外泌体分离纯度和回收率。反复的超速离心可以减少与外泌体共沉降的杂质从而提高纯度,但也会导致外泌体损失而降低回收率。尽管差速超速离心法目前被广泛认可和使用,但仍有许多缺点,比如依赖体积庞大且昂贵的仪器,处理过程耗时耗力(>4 h),所需初始样本量大,回收率低(<25%),聚集蛋白质污染,且超高速的离心力存在损伤外泌体结构从而降低生物活性的风险等^[10]^。

#### 1.1.2 密度梯度离心法

在差速超速离心法的基础上,发展了纯度更高且能分离特定外泌体亚群的密度梯度离心法。该方法基于外泌体密度(1.13~1.19 g/mL)与杂质密度的不同,使外泌体和杂质分别在不同密度梯度层溶液中达到离心力和浮力之间的平衡,从而实现与其他杂质颗粒的分离。在该方法中,外泌体样本可以“自下而上”(将样本与高密度介质混合加在离心管的底部,并在上方依次加入密度递减的梯度层,超速离心使密度小于周围介质的颗粒上浮,最终到达与其浮力密度相对应的密度层)或“自上而下”(梯度液的密度自上而下依次递增,将外泌体样本加到梯度顶部,超速离心使颗粒进入梯度并最终到达浮力密度平衡层)进行分离富集。蔗糖和碘克沙醇是常用的外泌体分离介质^[11]^。其中,碘克沙醇比蔗糖的渗透压低,更有助于保持外泌体的完整性和功能^[12]^。Van Deun等^[13]^评估了4种常用的外泌体制备方法(超速离心、OptiPrep密度梯度离心和2种市售试剂盒ExoQuick、Total Exosome Isolation Reagent)对外泌体产量、纯度、大小、形态以及蛋白质组和转录组含量的影响,结果表明分离方法的选择会严重影响外泌体群体的提取,从而影响组学特征,OptiPrep密度梯度离心法在外泌体纯度上优于其他3种方法,并揭示了独特的mRNA谱。然而,与传统的超速离心法相比,密度梯度离心法需要的分离时间更长(18 h^[11,14]^、62 h^[15]^、48~90 h^[16]^),并且对操作人员的技术要求更高。

基于“自下而上”和“自上而下”密度梯度离心法的外泌体分离示意图见图1^[17]^。

**图1:**
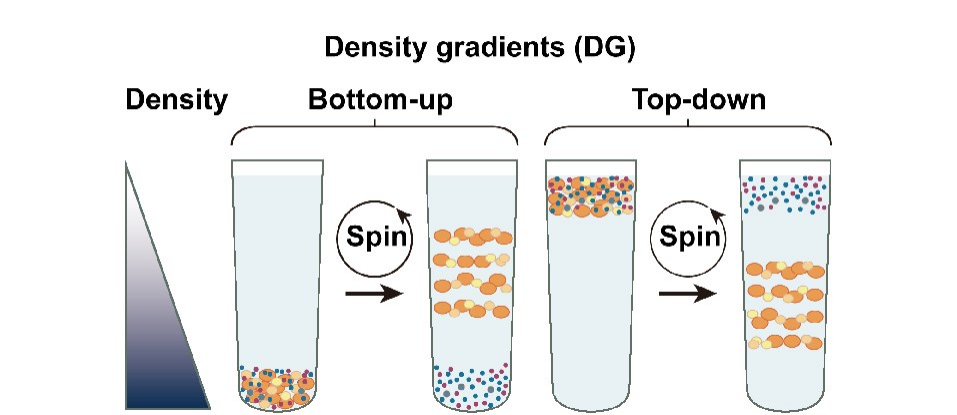
基于“自下而上”和“自上而下”密度梯度离心法的外泌体分离示意图^[17]^

### 1.2 聚合物沉淀法

聚合物沉淀法通过将亲水性聚合物添加到样本中,使外泌体周围的水分子被聚合物束缚,从而降低外泌体的溶解度并迫使外泌体在低速离心下聚集沉淀。聚乙二醇(PEG)是最常用于沉淀外泌体的聚合物^[18,19]^。Weng等^[20]^考察了聚合物种类、PEG相对分子质量和浓度以及离子强度对外泌体分离的影响,发现10% (g/mL) PEG (*M*_w_ 10 kDa)且不添加任何盐离子的富集效果最好。Kumar等^[21]^提出利用壳聚糖从多种生物体液中分离外泌体,壳聚糖是一种天然阳离子多糖,可通过与外泌体表面负电荷的磷脂膜相互作用促进沉淀, 但该方法依赖于在酸性条件(pH≈3)下溶解壳聚糖实现外泌体富集,中性条件下效率显著降低,且高盐洗脱(2 mol/L NaCl)可能破坏外泌体膜完整性或影响下游功能分析。目前市售的外泌体分离试剂盒大多基于聚合物沉淀原理,如ExoQuick(SBI)、Total Exosome Isolation kit(Thermo Fisher)和Exo-Prep(HansaBioMed)等。总之,聚合物沉淀法操作简便,外泌体收率高,具有可重复性,且不依赖大型设备,但是该方法分离的外泌体纯度不高,含有大量杂蛋白,且聚合物难以去除可能影响下游分析,使用过程中可考虑和其他分离方法结合以改进外泌体的制备。

### 1.3 基于尺寸的分离方法

基于尺寸的分离方法是指根据外泌体(30~200 nm)和其他杂质尺寸的差异对外泌体进行分离,主要包括超滤法、体积排阻色谱法、场流分离法等。

#### 1.3.1 超滤法

超滤法采用特定截留相对分子质量的多孔滤膜,尺寸大的外泌体颗粒被捕获在超滤膜上,而尺寸较小的杂质和多余液体穿过滤膜进入另一侧,从而实现外泌体的分离和浓缩^[22]^。Liu等^[23]^报道了一种基于纳米孔膜的外泌体总分离芯片(ExoTIC),使用200、100、80、50和30 nm孔径的滤膜模块串联,可以实现不同尺寸外泌体的分离。超滤法简单、经济且高效,但该方法容易使外泌体黏附在滤膜上造成损失,且容易堵塞膜孔导致外泌体产率下降和滤膜使用寿命缩短^[24]^。相比于死端过滤,切向流过滤技术提供了一种更有吸引力的选择。在切向流过滤中,流体沿滤膜平行方向流过,切向流速产生的剪切力大大减小了膜污染程度,从而有效减缓了滤饼层的堆积,提高了样品处理能力^[25]^。此外,由于过滤过程中保持较低的压力和剪切力,分离过程比较温和,有助于保持外泌体的结构和功能完整性。目前市售的配备有中空纤维膜的切向流装置,如KrosFlo KR2i和AKTA flux等,已成功用于外泌体的分离制备^[24,26]^。然而,这些装置仅依赖于尺寸筛分的单一分离机制,且膜孔径分布范围广,导致分离出的外泌体纯度不够。此外,这些设备在临床转化中存在操作繁琐、难以灭菌等困难。Hou等^[27]^提出了一种电场辅助切向流过滤系统(E-TFF),如图2所示,该系统结合20 nm滤膜的孔径筛分与基于电泳迁移的分离,实现了从细胞培养液中协同分离高质量外泌体。与超速离心法相比,E-TFF将外泌体纯度提高了1.4倍,产率提高了15.8倍,且用时仅为超速离心法的1/4,并证明了所制备外泌体的高生物活性,为外泌体的批量高效制备提供了有效的平台。

**图2:**
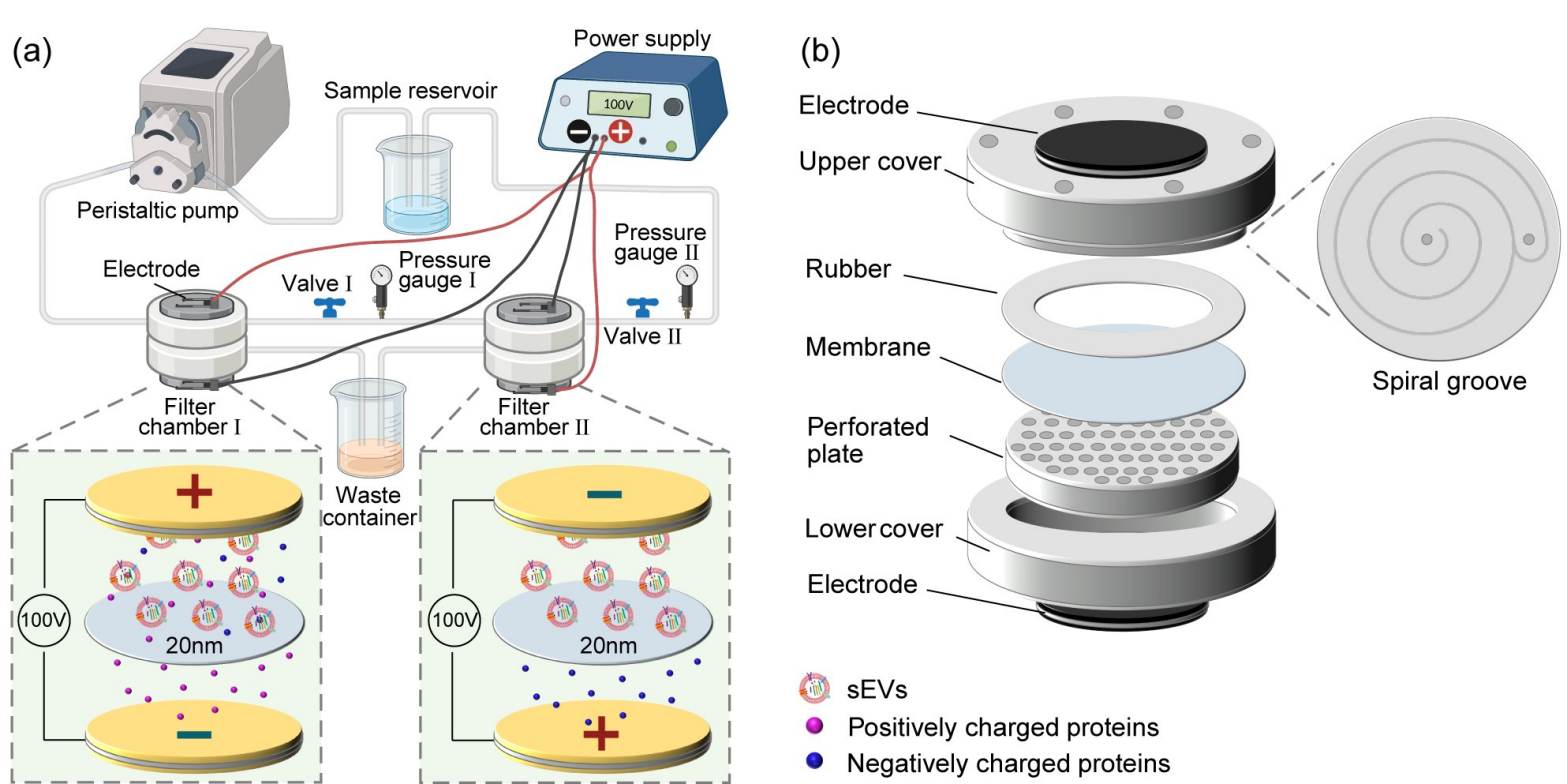
基于电场辅助切向流过滤系统的外泌体规模化制备示意图^[27]^

#### 1.3.2 体积排阻色谱法

体积排阻色谱法是采用多孔聚合物微球填充的色谱柱,利用外泌体与干扰蛋白的尺寸差异实现外泌体分离^[28]^。在色谱柱中,蛋白质和其他尺寸较小的污染物可以扩散到微球孔隙中,通过的路径较长从而洗脱时间长,而尺寸较大的外泌体可以快速通过并被直接洗脱,先于杂质流出色谱柱,从而实现外泌体与杂质的区分^[29]^。使用过程中,需考虑分离介质类型、孔径、外泌体与介质的相互作用、柱尺寸、柱填料以及流速等因素,以提高分离效率和分辨率。Guo等^[30]^通过优化柱填料孔径、填充体积、洗脱体积等,发展了二分式尺寸排除色谱法,只需1次大体积洗脱(总耗时约22 min)即可从血清、血浆等复杂体液中高效分离高纯度的外泌体。Zheng等^[31]^利用二维体积排阻色谱法实现了尿液来源外泌体大、中、小尺寸3个亚群的细分,并通过蛋白质组学技术分析了这3个亚群翻译后修饰蛋白表达谱的差异。目前,商品化的体积排阻色谱柱如SmartSEC Single for EV Isolation (SBI)、qEV (Izon)和Sepharose(GE Healthcare)等已被成功开发并广泛应用。体积排阻色谱法的独特优势在于,分离过程中剪切力很小,有利于保持外泌体的完整性和天然生物学特性,但该方法会稀释样本,且不适合大量样本处理,此外,该方法无法区分尺寸和外泌体相似的脂蛋白等非外泌体颗粒,导致分离纯度低,难以满足临床需求。

#### 1.3.3 场流分离法

场流分离是由Giddings等^[32,33]^于1966年提出的一种分离技术,指在样品流动方向上垂直施加力场,使流体中的颗粒在场力作用下实现基于大小的分离,该技术可以高分辨率地分离从几纳米到约100 μm大小的颗粒。在场流分离中,不同尺寸大小的颗粒在载液的层流作用下,同时受到垂直方向的外部力场和自身的布朗运动扩散的作用力,这两种驱动力的平衡使颗粒分布在通道内液流抛物线剖面不同高度的位置上,通道中心的流速较快,靠近通道内壁的流速相对较慢,从而达到分离的目的。根据所加外力场的类型,可以分为沉降场流分离、流场流分离、热场流分离、电场流分离等^[34]^。其中,非对称流场流分离技术被广泛应用于外泌体分离^[35⇓-37]^。在该方法中,分离通道的底壁被具有特定截留尺寸的半透膜代替,允许溶剂和小于截留尺寸的小分子透过,而保留大于截留尺寸的样品成分。Zhang等^[35,38]^利用非对称流场流分离结合紫外吸收与动态光散射的实时监测,分离得到了两种细胞外囊泡亚群(大囊泡,90~120 nm;小囊泡,60~80 nm)以及一类非膜性纳米颗粒(约35 nm),这3类亚群显示了不同的器官生物分布模式和生物功能。最近,Hu等^[37]^采用优化的不对称流场流分离结合密度垫超速离心法,从人血浆和血清中获得了高纯度、完整且脂蛋白污染极低的外泌体,并进一步利用蛋白质组学分析了血浆外泌体标志物。场流分离法具有快速、无标记、全自动、高重现性的优点,因不需要固定相及较小的外力,分离过程更加温和,与不同的检测器结合可用于所分离外泌体的进一步分析,应用不同类型的外力场可发挥尺寸分离与其他分离驱动力的协同效应。但该方法的局限性在于外泌体的回收率有待提高,不适用于大体积样品,且装置的复杂制作和操作在很大程度上限制了它们在生物医学研究中的应用和推广。

#### 1.3.4 反向富集法

基于外泌体和蛋白质杂质的尺寸差异,Li等^[39]^开发了第一个针对外泌体富集的反向富集策略(图3)。在该方法中,利用具有氧化石墨烯内核和聚醚砜外壳的材料(SNAPs),基于表面尺寸筛分以及氧化石墨烯对蛋白质的强大吸附特性,尺寸较小的杂质蛋白可以被氧化石墨烯不可逆地吸附在SNAPs内部,而由于SNAPs的表面孔径为10~40 nm,尺寸较大的外泌体被排除在材料外部,实现了尿液外泌体(4.91±1.01)×10^10^ 粒/μg蛋白质的富集,比超速离心、体积排阻色谱和PEG沉淀法高40.9~234倍,并实现了90.4%~93.8%的外泌体回收率。利用这种反向富集策略分离尿液外泌体并进行蛋白质组学分析,实现了免疫球蛋白A肾病患者和健康供体的成功区分。

**图3:**
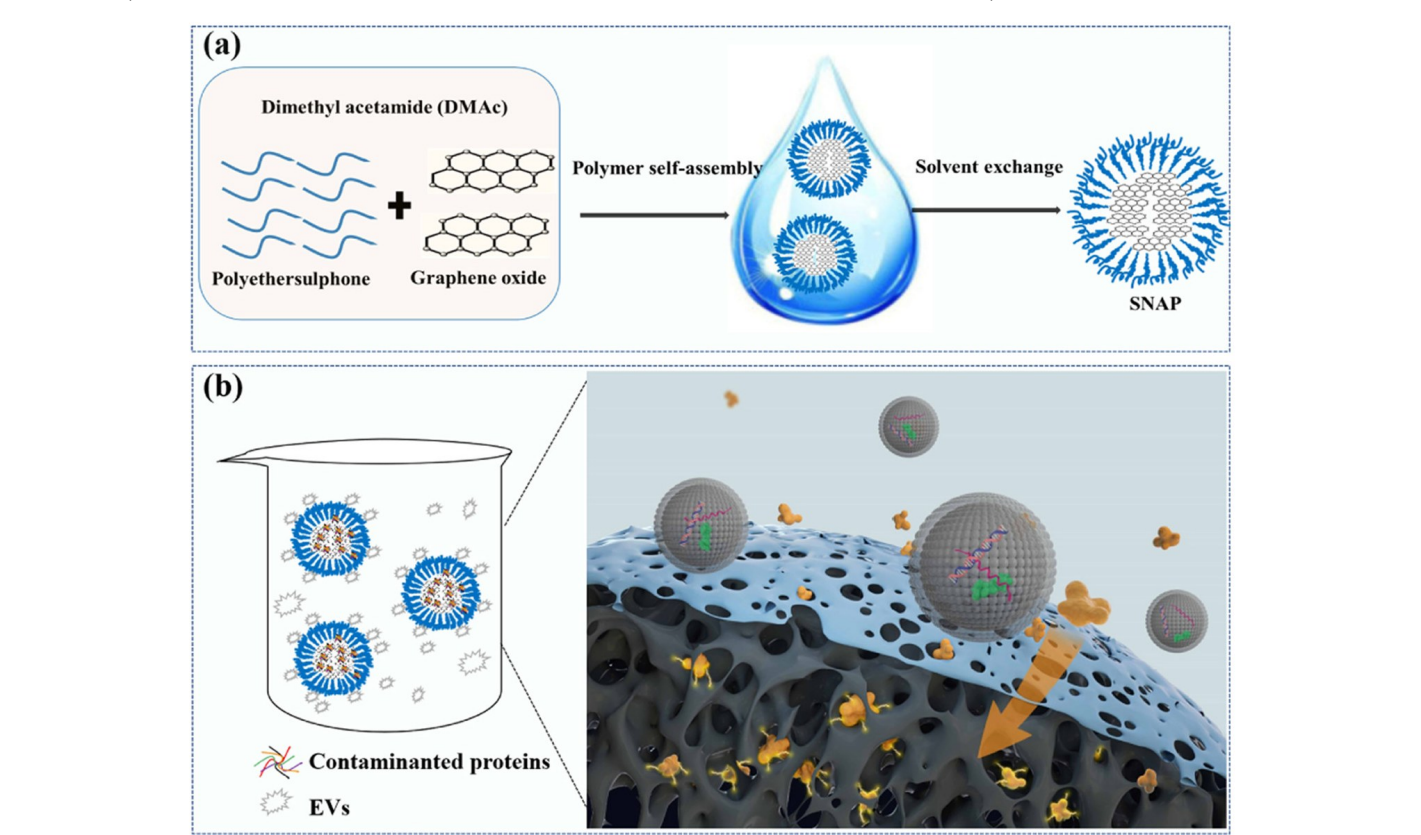
基于反向富集策略的外泌体分离方法示意图^[39]^

### 1.4 基于表面成分亲和的分离方法

近年来,利用与外泌体表面成分具有化学亲和力的成分,发展了许多新兴的外泌体分离方法。基于亲和的分离方法主要包括:针对外泌体表面蛋白的抗体^[40⇓-42]^、适配体^[43⇓-45]^,针对外泌体表面脂质的脂质探针^[46⇓-48]^,针对外泌体表面磷脂分子的磷脂酰胆碱(PC)亲和分子^[49]^、磷脂酰丝氨酸(PS)亲和分子^[50,51]^和对表面磷酸根具有高亲和力的钛(Ti)^[52]^,还有一些亲和肽^[53⇓-55]^等。

#### 1.4.1 抗体法

基于抗体的外泌体分离方法主要利用外泌体常见表面生物标志物的特异性抗体,如溶酶体相关膜蛋白3(CD63)^[40]^、白细胞分化抗原9(CD9)^[41]^和四跨膜相关蛋白1(CD81)^[42]^以及癌症特异性标志物的抗体,如上皮细胞黏附分子(EpCAM)^[56]^、表皮生长因子受体(EGFR)^[57]^和磷脂酰肌醇蛋白聚糖-1(GPC-1)^[58]^等,实现肿瘤来源外泌体的分离。最近,Li等^[42]^发展了一种外泌体识别和检测生物芯片(EVID-biochip),通过将CD81抗体和具有抗污染能力的两性离子修饰在磁球上,将磁球和血清样品等速注入微流控芯片的蛇形通道中,实现了帕金森病患者血清中外泌体的高效分离。Wang等^[40]^将CD63修饰在具有高表面积和优异分散性能的纤维素纳米纤维上,实现了高达86.4%的外泌体捕获效率,将其应用于分离血浆中外泌体并分析癌症相关蛋白和microRNA,为液体活检中的肿瘤诊断提供了有希望的标志物。基于抗体的外泌体分离方法简单、快速,具有高特异性,但存在试剂昂贵,步骤繁琐、处理样本量小等局限性,不适合高通量分离,此外抗体-抗原结合的强亲和力也使外泌体解离以进行后续功能分析具有挑战性。

#### 1.4.2 适配体法

除了基于抗体的免疫亲和方法,还发展了适配体作为抗体的替代品用于外泌体的靶向分离捕获。适配体是一类通过指数富集的配体系统进化技术(SELEX)筛选得到的具有特异性分子识别能力的单链寡核苷酸。目前用于外泌体分离的核酸适配体的筛选主要以外泌体表面蛋白质为靶标。适配体通常被修饰在不同的材料或界面,如磁性Ti_3_C_2_复合材料^[43]^、纳米球^[44]^、微流控芯片表面^[45]^、磁球^[59]^、碳布^[60]^等,用于外泌体的高效捕获。Cui等^[43]^制备了一种CD63适配体功能化的磁性Ti_3_C_2_复合材料(Fe_3_O_4_@Ti_3_C_2_@PEI@DSP@aptamer@FAM-ssDNA)用于同时富集和检测外泌体,CD63适配体识别和捕获外泌体后进行磁分离,最后通过切割二硫键释放外泌体,实现了保持结构和功能完整性的外泌体高效富集。Zhang等^[44]^报道了一种使用CD63适配体功能化的聚多巴胺纳米球(SIMPLE)用于快速分离和高灵敏度多色彩视觉检测外泌体的新方法,通过合成170 nm的适配体功能化的聚多巴胺纳米球,可以选择性结合外泌体表面的特定蛋白质,两者的结合使外泌体的总体尺寸增加,从而允许通过滤膜(孔径200 nm)快速分离外泌体。与抗体相比,适配体具有稳定性高、易于位点特异性修饰、空间位阻低、合成可控、成本低等优点。但该方法的局限性在于,目前发展的适配体只能识别外泌体表面的特异性蛋白,其识别和捕捉效果取决于该蛋白质在外泌体表面表达的含量,由于外泌体的高度异质性,并不是所有外泌体都在表面表达同一种蛋白质,因此导致适配体的结合性能降低。

#### 1.4.3 基于脂质分子亲和的分离

外泌体表面的磷脂双分子层也是亲和捕获的重要目标。脂质探针和外泌体脂质膜的强疏水相互作用是实现外泌体有效分离的途径之一。Wan等^[61]^报道了一种脂质纳米探针(LNP)系统,利用生物素标记的1,2-二硬脂酰-snglycero-3-磷酸乙醇胺(DSPE)通过疏水作用插入外泌体膜,随后通过与亲和素磁球的结合,实现细胞培养液和血浆中外泌体的快速分离。Feng等^[62]^发展了一种两亲分子-树突状分子超分子探针(ADSP),如图4所示,利用具有两亲性的两性霉素B功能化到高度分支的树突状分子上,可以有效地插入外泌体膜,通过探针与外泌体脂质双分子层之间的多价相互作用实现外泌体的高效捕获,将ADSP涂覆在硝化纤维素(NC)膜上,实现了外泌体的原位捕获和蛋白质检测。最近,Weerakkody等^[46]^发展了用于细胞外囊泡亚群快速高纯度分离和大小特异性富集的光敏纳米脂质探针,探针中DSPE可确保有效的外泌体捕获,光敏连接剂在光照下发生裂解促进外泌体以接近天然的形式释放,实现了1 h内从复杂的生物培养基中分离出高纯度的外泌体,并将其分离成基于大小的亚群,比超速离心具有更高的效率和纯度。Pan等^[48]^报道了一种快速高效的外泌体分离平台(EV-FISHER),由金属-有机框架(MOF)结合可断裂脂质探针(P ${O_{4}}^{3-}$ -DNA-胆固醇,PSDC)组成,胆固醇用于捕获外泌体,低速离心实现富集,随后加入脱氧核糖核酸酶Ⅰ (DNase Ⅰ)水解PSDC中的DNA以分离外泌体。胆固醇对外泌体膜的高亲和力结合MOF的优势使EV-FISHER在时间、分离效率和分离条件方面优于超速离心法。与外泌体磷脂膜中磷酸根具有螯合作用和高亲和力的钛(Ti)也是实现外泌体分离的有效途径。Pan等^[63]^利用二氧化钛微球高选择性纯化血浆中的外泌体,与超速离心法相比,该方法可将分离过程缩短至20 min,从100 μL血浆中分离得到约10^8^个外泌体粒子,并将其用以描述糖尿病视网膜病变的代谢特征。

**图4:**
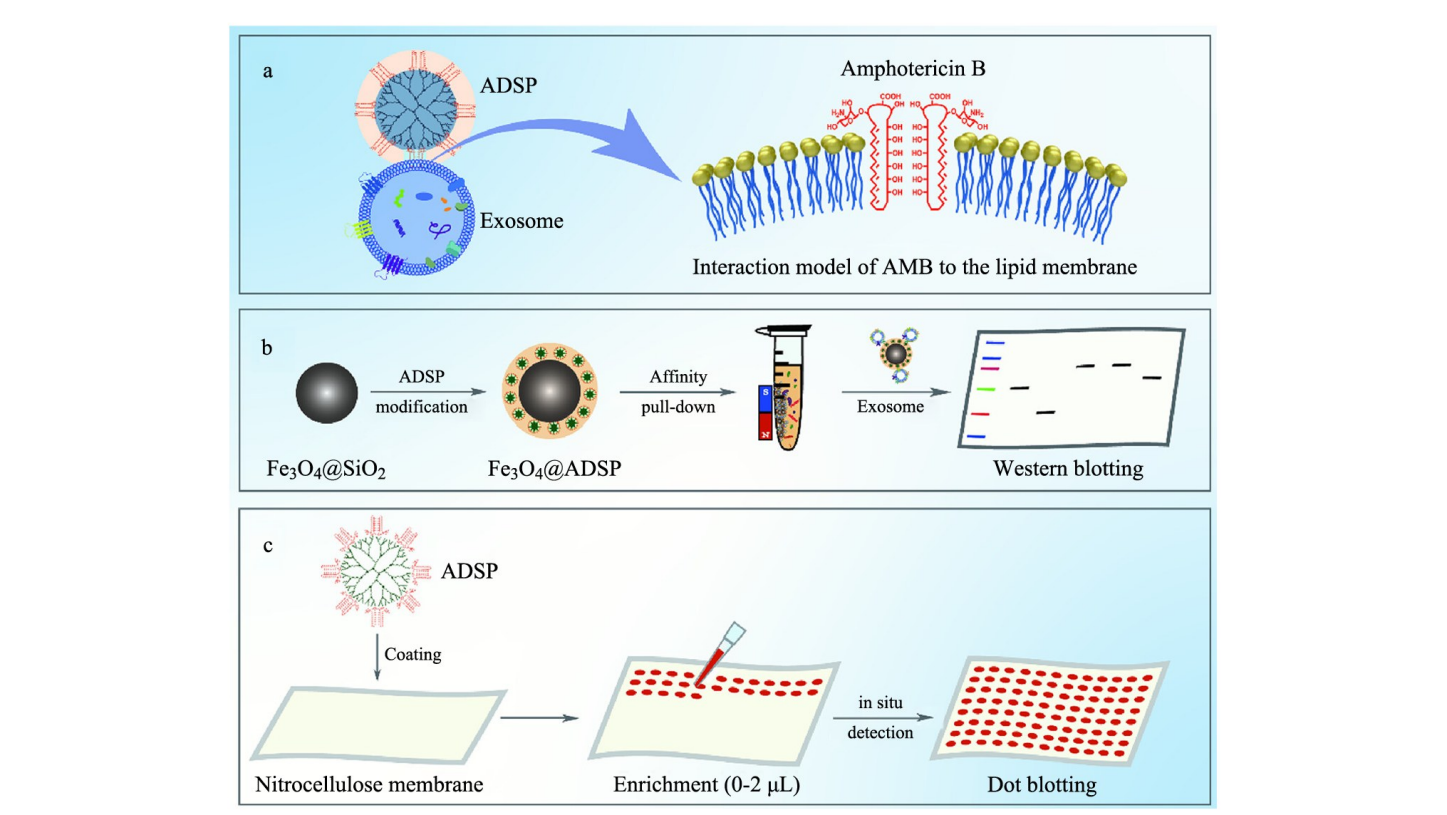
基于两亲分子-树突状分子超分子探针的外泌体分离方法示意图^[62]^

针对外泌体表面特定的磷脂分子也发展了很多新兴的分离方法。磷脂酰丝氨酸(PS)和磷脂酰胆碱(PC)是外泌体膜上两类主要的磷脂分子^[64]^。T细胞免疫球蛋白和黏蛋白结构域蛋白4(Tim4)是PS分子的天然受体,对PS分子有高亲和力^[65]^。Zhou等^[50]^发展了一种PS印迹材料,通过与PS亲水头部基团特异性结合,实现了人尿液外泌体的高效和高特异性分离。Li等^[49]^基于外泌体膜上的PC和修饰在磁球上的磷酸胆碱(CP)之间的可逆两性离子配位,发展了一种快速(<30 min)、收率和纯度超过90%、可适用于多种生物体液(如血清、尿液和唾液)的外泌体分离方法,将该方法与蛋白质组学相结合,鉴定出外泌体上的一组差异表达蛋白作为潜在的结肠癌生物标志物(图5)。

**图5:**
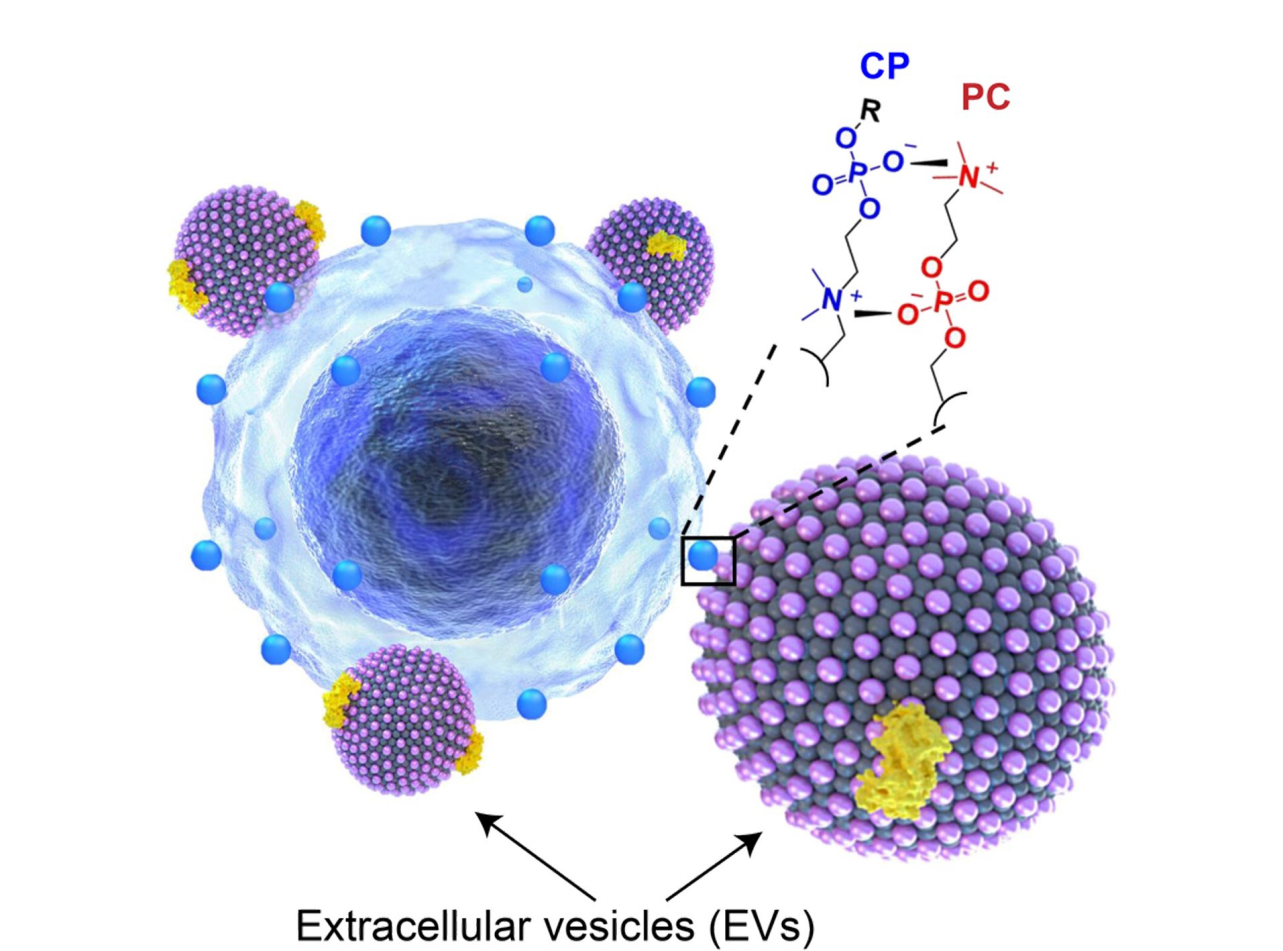
基于PC-CP两性离子配位的外泌体分离方法示意图^[49]^

#### 1.4.4 基于亲和多肽的分离

对外泌体表面具有特异性亲和力的多肽也被用于外泌体的分离。Sun等^[53]^利用一种带有高正电荷的穿膜钛八精氨酸 $R_{8}^{+}$ 和Ti(Ⅳ)协同分离微量培养液中外泌体并进行磷酸蛋白质组学分析,将其应用于小体积脑脊液监测原发性中枢神经系统淋巴瘤患者的化疗结果,为临床应用提供了工具。Zong等^[66]^提出一种双开关低pH插入肽(D-S pHLIP)系统,能特异性地插入循环肿瘤微环境来源的外泌体膜中,再结合磁分离策略,选择性地从血清中分离富集循环肿瘤微环境来源的外泌体。Salerno等^[54]^开发了一种BPt肽功能化中空纤维膜组件,BPt肽是一种基于静电和疏水相互作用选择性结合纳米囊泡膜的肽序列,实现了人心脏祖细胞培养液中高纯度外泌体的分离富集。

随着外泌体领域的快速发展,新型的分离方法不断推陈出新。表1总结了目前常用的外泌体分离方法的原理、优缺点及适用性。迄今为止,仍然没有普遍适用的外泌体分离方法,需要根据特定的研究目的选择相应的适用方法。另外,多种方法结合使用也是提高外泌体分离质量的重要发展趋势。

**表1:** 外泌体分离方法的原理、优缺点及适用性

Method	Principle	Advantages	Disadvantages	Refs.
Differential ultracentrifugation	density-based isolation	gold standard method; relatively high purity	time-consuming (>4 h); low recovery; protein aggregation; damaged exosome structure	[9,10]
Density gradient ultracentrifugation	density-based isolation	high purity	very time-consuming (>18 h); high operating requirements; low recovery	[11-13]
Polymer-based precipitation	hydrophobic effect	simple operation; high recovery	low purity; lack of specificity; difficult to remove polymer	[18,20,21]
Ultrafiltration	size-based isolation	simple operation; economical and efficient	filter membrane easily clogged; low purity; large sample volume	[23,24,27]
Size-exclusion chromatography	size-based isolation	simple and efficient; maintaining the integrity and biological activity of exosomes	low processing throughput; low purity	[28,30]
Field flow fractionation	size-based isolation	gentle and fast separation process	time consuming; low recovery; requires specialized equipment; high operating requirements	[35-37]
Chemical affinity	affinity-based isolation	high purity and specificity	high cost; not suitable for large-scale sample processing	[40,45,46,49]

## 2 外泌体分离技术在临床诊疗中的应用

外泌体具有广泛的生物学功能,并参与多种生理和病理过程,在正常和病理条件下均可从细胞中释放,它们在细胞通讯中的关键作用使其成为疾病生物标志物、治疗药物和药物递送系统的有潜力的工具^[6]^。

### 2.1 外泌体疾病诊治标志物筛选

液体活检是指从患者的各种生物体液中取样和分析,以获取与疾病相关的信息。相比于组织活检,液体活检具有非侵入性、早期诊断、可动态监测等优势。循环肿瘤细胞、循环肿瘤DNA、外泌体等都是液体活检的分析对象^[67]^。外泌体几乎从所有活细胞中分泌,含有丰富的蛋白质、核酸、脂质、代谢物等物质,大量存在于生物体液中,并且外泌体的脂质双分子层使它们能够稳定地携带和传递来自其母体细胞的重要生物信息。因此,通过对血浆、血清、尿液、唾液等生物体液进行外泌体的分离分析,有望捕获复杂的细胞内分子特征,监测正常生理或病理状况时的细胞状态,使外泌体成为一个有吸引力的疾病诊断治疗标志物的储存库。其中,外泌体蛋白是重要的标志物来源,基于色谱-质谱联用的蛋白质组学技术为外泌体生物标志物的发现提供了强大的技术手段^[4]^。外泌体蛋白质标志物的研究主要包括3个阶段:(1)发现阶段:首先要确保分析对象为高纯度外泌体,以保证来源的真实有效性,随后进行深度覆盖的蛋白质组学分析,对外泌体中的蛋白质进行定性和定量,最后进行统计分析以筛选潜在的生物标志物;(2)验证阶段:评估一些最有希望的候选标志物,以验证之前的结果;(3)确认阶段:通过分析扩大或独立队列的相同疾病个体样本,确认生物标志物在疾病中的效用^[68]^。

Zheng等^[69]^发展了串联体积排阻色谱法用于快速高效分离人血浆外泌体,并结合质谱指纹图谱及人工神经网络模型,实现对乳腺癌、胰腺癌等不同癌症类型的区分,实现了80.0%的诊断准确率。针对外泌体表面肿瘤生物标志物的信号强度易被抑制,需引入信号放大策略的问题,Yan等^[70]^发展了针对胰腺癌外泌体生物标志物GPC1进行原位信号放大定量检测的分析方法,采用抗GPC1抗体和质量标签修饰的金纳米颗粒,将大分子GPC1信号转换为有机低聚物的质量标签信号,实现了胰腺癌患者和健康对照的高精度区分(*P*<0.0001)。针对缺乏高效、高通量的唾液生物标志物蛋白质组学分析方法,Shen等^[71]^设计了一种两亲性树枝状聚合物超分子探针(SEASP)阵列,可有效富集和原位检测唾液外泌体蛋白质生物标志物,结合基于质谱的蛋白质组学分析,筛选出差异表达蛋白质作为哮喘生物标志物,使用SEASP阵列通过临床样本验证了蛋白质组学初步筛选结果,发现MUC5B和HNNPU是哮喘患者唾液外泌体的潜在生物标志物。针对外泌体内容物的低表达和单个生物标志物诊断能力不足的问题,He等^[72]^提出了一种外泌体蛋白质和RNA的多重共检测平台(Co-PAR)。Co-PAR采用一对抗体-DNA探针特异性识别相同的靶蛋白并构建双链DNA序列,将靶蛋白检测转化为双链DNA序列的定量,应用逆转录聚合酶链反应同时定量同一外泌体样品中的靶RNA。使用Co-PAR分析了3种蛋白质标志物(CD63、GPC-1、HER2)和3种RNA标志物(snRNA U6、GPC-1 mRNA、miR-10b)在3种胰腺细胞系和30份人血浆样本的外泌体上的共表达,6种生物标志物组合的诊断准确率达到92.9%。针对阿尔茨海默病(AD)的无创和高效诊断,Cai等^[73]^使用邻近条形码试验(PBA)在5种易于获得的体液中进行了单个外泌体水平的外泌体表面蛋白的多重分析,使用PBA测定分析了来自AD小鼠和人类受试者的鼻腔、口腔、眼部冲洗液以及尿液和血液各种类型体液中的近200种外泌体膜蛋白,结合机器学习预测模型,发现尿液外泌体蛋白在小鼠和人类中都表现出最高的诊断潜力,单外泌体分析进一步揭示了测试体液中与AD相关的外泌体亚群,具有特征蛋白PLAU、ITGAX和ANXA1的尿液外泌体亚群以88%的准确率诊断AD,表明来自无创体液(尤其是尿液)的外泌体及其亚群是AD的潜在诊断生物标志物。

### 2.2 外泌体在疾病治疗中的应用

外泌体的治疗潜力主要体现在两个方面:(1)干细胞来源的外泌体以旁分泌机制发挥免疫调节和促再生能力,且具有高生物活性、低免疫原性、高安全性和易于存储等优点,有望成为神经退行性疾病、心血管疾病和癌症等疾病的新型治疗剂;(2)天然的外泌体生物相容性好,可以被改造装载治疗药物,靶向表面受体或配体递送到目标受体细胞,且易穿透生物屏障,是优良的药物递送载体。

#### 2.2.1 干细胞来源外泌体作为治疗剂

间充质干细胞(mesenchymal stem cells, MSCs)是一种多能干细胞,具有自我更新和多向分化能力。基于MSCs的疗法已成为一种有吸引力的策略,可通过免疫调节和组织修复来治疗人类疾病。然而,越来越多的研究数据表明,MSCs发挥治疗效果的关键机制之一是MSCs释放的外泌体、细胞因子等分泌组介导的旁分泌作用^[74,75]^。外泌体通过将信息传递给受损细胞或组织来参与组织再生,并发挥类似于MSCs的生物活性。据报道,MSCs来源的外泌体可以减少心肌缺血/再灌注损伤^[76]^,减轻肾脏炎症^[77]^,缓解肺纤维化^[78]^,修复伤口损伤^[79]^,发挥神经保护作用^[80]^等。与细胞疗法相比,外泌体更易于管理且更安全,目前没有已知的免疫排斥或肿瘤发生的风险^[81,82]^。

针对肾单侧缺血再灌注损伤,Guo等^[83]^报道了一种高质量外泌体的高效制备技术,通过生物打印微纤维间充质干细胞培养使外泌体产量增加约1000倍,外泌体的体外给药通过减少上皮-间充质转化、细胞外基质沉积和铁死亡,增强了NRK-52E细胞的增殖和迁移,并减轻了缺氧/复氧诱导的损伤,结合外泌体的尾静脉注射,发现小鼠肾脏中肾功能的改善和胶原蛋白沉积减少,表现出增强的治疗效果。基于人脐带间充质干细胞来源外泌体强大的免疫抑制和抗氧化特性,Wang等^[84]^研究了人脐带间充质干细胞衍生外泌体对白癜风的治疗效果,发现外泌体给药显著改善了白癜风小鼠模型的色素脱失,并深入探究了治疗机制,发现携带miR-132-3p和miR-125b-5p的外泌体可以通过分别靶向Sirt1和Bak1诱导Treg细胞分化并抑制氧化应激诱导的黑色素细胞凋亡,从而保护黑色素细胞免受氧化和免疫破坏,并最终缓解白癜风的进展。Kaur等^[85]^将骨髓间充质干细胞来源的外泌体用于治疗自身免疫性葡萄膜视网膜炎小鼠模型,发现间充质干细胞外泌体可以直接抑制视网膜反应性T细胞向眼睛的浸润,从而阻止自身免疫性葡萄膜视网膜炎小鼠的疾病进展。

#### 2.2.2 外泌体作为药物递送载体

外泌体可被修饰以靶向特定细胞,能负载多种药物,并具有优越的穿透各种组织屏障的能力,是优良的天然药物递送载体。相比于脂质纳米颗粒(LNP),天然的外泌体表现出更低的免疫原性和更高的生物相容性^[86]^。外泌体可以装载不同的物质,包括DNA、RNA、蛋白质、多肽、抗体、小分子药物、成像探针等。外泌体装载策略主要有两种:(1)预装载:在外泌体被分离之前通过共孵育、转染等方式将物质装载到亲本细胞中,随后亲本细胞分泌出装载有特定物质的外泌体;(2)后装载:是指将外泌体分离后,通过共孵育、电穿孔、超声、挤出、冻融循环、转染等方式将物质直接装载进外泌体^[87]^。基于外泌体的给药具有很高的灵活性和相容性,可以通过静脉、皮下、鼻内、腹腔注射和口服等方式给药^[88]^。为了将目标药物递送到特定的组织或细胞,还需要赋予天然外泌体靶向能力,通常利用转染或化学修饰等方法将靶向分子组装在外泌体膜上,通过配体-受体结合、pH梯度、电吸引或磁场被引导至靶细胞^[88]^。

针对可编程核酸酶Cas9难以体内递送实现有效的T细胞靶向,Stranford等^[89]^报告了一套用于细胞基因工程的方法,工程化的细胞分泌的外泌体通过蛋白质标签主动装载蛋白质货物,并通过高亲和力的T细胞靶向结构域和融合性糖蛋白来实现外泌体的靶向传递,产生的外泌体可以在细胞之间自然地包裹和转移蛋白质和核酸,从而在不需要化学修饰的情况下将生物制剂靶向递送到T细胞。针对多种遗传药物难以精确地递送到胰腺导管腺肿瘤导致治疗反应不佳的问题,Chiang等^[90]^报道了携带高负荷治疗性RNA的双靶向外泌体(dtEV)用于有效抑制小鼠的大型胰腺导管腺癌肿瘤,利用非对称细胞电穿孔技术,将编码多种基因的质粒转染细胞,使外泌体表面装载可以靶向胰腺肿瘤组织的CD64蛋白,另外CD64也可以结合ROR1抗体靶向多种肿瘤细胞从而实现双靶向,使外泌体内部装载TP53抑癌基因编码的mRNA,或靶向KRAS^G12D^突变的siRNA,实现有效的胰腺癌治疗。针对目前mRNA递送存在的脱靶效应和毒性等问题,Liu等^[91]^通过电穿孔将IL-12 mRNA装载到人胚胎肾细胞来源的外泌体(HEK-Exo)中,得到IL-12 mRNA负载的外泌体(IL-12- Exo),通过吸入方式将载药外泌体直接输送到肺部,实现靶向递送和更少的全身副作用,并增强肺肿瘤小鼠模型的全身免疫,实现肺肿瘤抑制并有效防止肿瘤再复发。

## 3 总结与展望

近年来,外泌体领域高速发展,越来越多的重要研究进展加深了人们对外泌体结构和功能的认识。外泌体在介导细胞间通讯、维持机体平衡和提供免疫稳态等方面的重要作用使其成为临床诊疗的重要工具。然而,目前针对外泌体的基础研究和临床应用仍然存在很多挑战。首先,由于外泌体的复杂性和异质性,缺乏标准化的外泌体分离方法使研究结果具有高度可变性,阻碍了不同实验室和临床环境之间结果的比较和可重复性。目前的分离方法普遍存在纯度不够、收率低、通量低、难以分离特定亚群等问题,严重制约了外泌体领域的发展。其次,缺乏便于临床使用的技术要求低、效果稳定、高效率、成本低的外泌体分离方法。由于临床环境的特殊性,目前常用的超速离心等经典方法具有耗时耗力、需求样本量大等缺陷不适于临床转化,免疫亲和等方法可用于临床珍贵微量样本的外泌体分离,但是存在成本高、回收率低、操作要求高等不足,制约了临床环境中样本的分析。另外,缺乏规模化制备外泌体的稳健方法。随着外泌体在临床疾病治疗方面的快速进展,亟需发展可重复和可扩展的批量制备高质量外泌体的方法,满足临床大量外泌体制剂的需求。总的来说,外泌体研究的进展将大大提高对外泌体生物学功能和组成成分的理解,进而有助于将外泌体的研究转化为有效的诊断和治疗策略,为精准医学和个性化治疗提供新的手段。
